# Prevalence, characterization, and implications of methicillin-resistant *Staphylococcus aureus* (MRSA) in ready-to-eat foods from Delta, Nigeria: a concern for consumer safety

**DOI:** 10.1093/sumbio/qvae007

**Published:** 2024-04-22

**Authors:** Abeni Beshiru, Brenda O Isichei-Ukah, Kate E Uwhuba, Bright E Igere, Etinosa O Igbinosa

**Affiliations:** Department of Microbiology, College of Natural and Applied Sciences, Western Delta University, 331101 Oghara, Delta State, Nigeria; Applied Microbial Processes and Environmental Health Research Group, Faculty of Life Sciences, University of Benin, Benin City 300283, Nigeria; Department of Microbiology, College of Natural and Applied Sciences, Western Delta University, 331101 Oghara, Delta State, Nigeria; Department of Microbiology, Dennis Osadebay University, 320231 Anwai, Asaba, Delta State, Nigeria; Applied Microbial Processes and Environmental Health Research Group, Faculty of Life Sciences, University of Benin, Benin City 300283, Nigeria

**Keywords:** Ready-to-eat foods, MRSA, antibiotic resistance, food safety, Nigeria

## Abstract

Ready-to-eat (RTE) foods are susceptible to contamination with methicillin-resistant *Staphylococcus aureus* (MRSA), presenting significant health risks to consumers. This study aimed to isolate, identify, and characterize MRSA from RTE foods in Delta, Nigeria, and assess their implications for consumer safety. Four hundred RTE food samples were collected from food outlets, and MRSA presence was determined using oxacillin resistance screening agar supplemented with polymyxin B and oxacillin. Polymerase chain reaction confirmed and characterized MRSA isolates for virulence potentials and antimicrobial resistance genes. Out of the 400 samples, 57(14.25%) tested positive for MRSA. The prevalence of virulence genes varied, with Panton-Valentine Leukocidin (*pvl*) detected in 40.51% of isolates, along with the detection of several staphylococcal enterotoxin genes. Antimicrobial resistance genes, including tetracycline (*tetM*, 43.04%), erythromycin (*ermC*, 32.91%), and methicillin (*mecA*, 100%; *mecC*, 29.11%) were detected. Staphylococcal cassette chromosome *mec* (*SCCmec*) typing revealed diverse profiles, with type V being predominant (32.9%). MRSA isolates exhibited resistance to multiple antibiotics, with 83.54% of them classified as multidrug-resistant. Extracellular virulence factors were common, with strong biofilm producers observed in 69.62% of isolates. These findings underscore the complexity of MRSA contamination in RTE foods, highlighting the need for enhanced surveillance and control measures to safeguard public health.

**Sustainability Statement**

The scientific findings presented in this study on the prevalence and characterization of methicillin-resistant *Staphylococcus aureus* (MRSA) in ready-to-eat (RTE) foods in Delta State, Nigeria, hold significant implications for sustainability across multiple fronts. Firstly, addressing the issue of food safety, particularly in RTE foods, is vital for achieving Goal 2: zero hunger and Goal 3: good health and well-being. By identifying MRSA contamination in RTE foods and assessing its antibiotic resistance (AMR) and virulence factors, this research contributes to ensuring food security and promoting public health. Preventing foodborne illnesses resulting from MRSA contamination aligns with the United Nation's (UN) aim to ensure access to safe, nutritious, and sufficient food for all, thereby improving overall health and well-being. The scientific observations from this study contribute to addressing key sustainability goals related to ending hunger, promoting good health and well-being, ensuring sanitation, and combating AMR and its environmental impacts. By addressing food safety concerns and promoting sustainable food production practices, this research contributes to building resilient and sustainable societies in line with the UN Sustainable Development Goals.

## Introduction

Ready-to-eat (RTE) foods are integral to modern dietary habits, offering unmatched convenience without requiring additional preparation. From sandwiches and salads to deli meats and prepackaged meals, RTE foods are easily accessible across various food outlets, including supermarkets and restaurants. These foods, defined as immediately consumable for ensured food safety, encompass a wide array of items such as salads, cooked meats, desserts, and precooked dishes intended for cold serving, including shelf-stable, refrigerated, or frozen products (EFSA Panel on Biological Hazards et al. 2018). Despite their widespread popularity, RTE foods present unique food safety challenges, being consumed without further processing, rendering them susceptible to pathogens like *Staphylococcus aureus* (Esemu et al. 2023). *S. aureus*, a Gram-positive bacterium frequently found on animal and human skin and mucous membranes, exhibits remarkable versatility as a pathogen, leading to infections spanning from mild to life-threatening (Shoaib et al. 2023). Despite its commensal presence in up to 80% of healthy individuals, *S. aureus* continues to be a primary contributor to soft tissue and skin infections, often leading to complications like necrotizing pneumonia and septic arthritis. Its toxin arsenal includes toxic shock syndrome toxin 1 (TSST-1), Panton-Valentine Leukocidin (PVL) toxin, hemolysins, staphylococcal enterotoxins (SEs), and exfoliative toxins (ETs) (Linz et al. 2023). PVL induces cytotoxicity, causing tissue damage and various infections. At the same time, SEs lead to food poisoning, and ETs and TSST-1 cause staphylococcal scalded skin syndrome and toxic shock syndrome (TSS), respectively (Ahmad-Mansour et al. 2021).

*S. aureus* infections pose significant challenges due to antimicrobial resistance (AMR), with methicillin-resistant *Staphylococcus aureus* (MRSA) (Abebe and Birhanu ). MRSA resistance complicates treatment strategies and raises global health concerns, as underscored by the World Health Organization's designation of MRSA as a high-priority AMR pathogen (Tacconelli et al. 2018, Mancuso et al. 2021). Recent attention has turned to MRSA contamination in RTE foods, with associated outbreaks emphasizing the need for stringent monitoring and control measures across the food supply chain (Wang et al. 2023). However, research on MRSA prevalence and attributes in RTE foods, particularly in regions with high consumption rates, still needs to be completed. MRSA's impact extends beyond healthcare, affecting communities, hospitals, and livestock, highlighting the urgency of addressing this menace. Recent epidemiological data reveal significant mortality and morbidity burdens from MRSA infections (Hasanpour et al. 2023). The acquisition of methicillin resistance genes (*mecA* or *mecC*) enables resistance through penicillin-binding protein 2a (PBP2a) activity, facilitated by the *mec* operon within the staphylococcal cassette chromosome *mec* (*SCCmec*) (Miragaia 2018).

Epidemiologically, MRSA is categorized into distinct groups: community-associated MRSA (CA-MRSA), livestock-associated MRSA (LA-MRSA), and hospital-associated MRSA (HA-MRSA) (Kasela et al. 2023). HA-MRSA emerged in 1961, transitioning into the community by 1990 before infiltrating healthcare settings, while LA-MRSA is historically linked to animals, transmitting from them to humans (Shoaib et al. 2023). Genetic variations characterize MRSA across classifications. CA-MRSA often harbours *SCCmec* types V or IV, with potent toxin production like PVL, whereas HA-MRSA displays larger *SCCmec* types I-III and heightened AMR (Funaki et al. 2019). Livestock-associated MRSA presents unique co-resistance to non-β-lactam antimicrobials, with *SCCmec* elements differing from other MRSA genotypes (Wu et al. 2019). Some LA-MRSA lacks crucial virulence factors like PVL (Karampatakis et al. 2021). Reports indicate MRSA strains in various food sources, implying food as a reservoir for dissemination, highlighting the diverse origins of food products, where different MRSA strains may be found across regions (Wu et al. 2019, Esemu et al. 2023, Wang et al. 2023).

*S. aureus* gastroenteritis is a prevalent global foodborne illness, with increasing resistance to beta-lactam antibiotics compounding public health concerns (Grudlewska-Buda et al. 2023). Investigating MRSA prevalence in food is vital to assess contamination risks and human infection sources. Antibiotic use in livestock amplifies multidrug-resistant (MDR) bacteria, transferring risks to humans via contaminated meat (Almansour et al. 2023). Understanding MRSA transmission in the food chain is critical for effective AMR strategies and public health protection. Apart from direct transmission from animals, MRSA can spread via contaminated foods (da Silva et al. 2020). Individuals in contact with animal reservoirs or consuming MRSA-contaminated foods may become carriers, facilitating community spread. Focusing on animal-derived foods, particularly RTE foods with extensive manual handling, is crucial for sampling and testing efforts. Despite recognizing the significance of understanding MRSA prevalence in food, Nigeria needs more substantial data. Investigating MRSA in retail food samples from Delta State, Nigeria, aims to fill this gap. Delta State's diverse culinary landscape offers various RTE options, which are popular for convenience despite lingering safety concerns. More resources exacerbate food safety challenges. This study's primary aim is to assess MRSA prevalence and characteristics in RTE foods in Delta State, informing interventions to mitigate contamination risks and enhance food safety. Addressing this knowledge gap will support evidence-based policies to reduce foodborne illnesses and AMR, ultimately enhancing public health in Nigeria.

## Materials and methods

### Sample collection

A cross-sectional study was conducted to evaluate MRSA in RTE foods within Delta State. The research specifically targeted RTE foods originating from Delta State, Nigeria. Sample size determination followed a formulaic approach as outlined below:


\begin{eqnarray*}
{\rm Sample}\left({\rm N}\right) = \frac{{\left( {{{\rm Z}_{1 - \propto /2}}} \right)^2\,\, {\rm P}\left( {1 - {\rm P}} \right)}}{{{\rm d}^{2}}}
,
\end{eqnarray*}

Where:

*Z_1-α/2_* = Standard normal variant at 5% type I error (*P* < 0.05); *P* = Expected prevalence based on previous studies [32%, 8% (Contreras et al. 2015); 22% (Islam et al. 2019); 27% (Sergelidis et al. 2012); 12.5% (Yang et al. 2016); 46% (Adzitey et al. 2020)]; *d* = Absolute error or precision (set at 5%). A total of 400 RTE food samples were randomly procured from various food outlets across Delta State, Nigeria, during April 2021, utilizing sterile universal containers. All samples were carefully sealed and transported in ice boxes to the laboratory for subsequent analysis.

### Laboratory enumeration and isolation of MRSA from the RTE food samples

Twenty-five (25) grams of RTE food samples were weighed and homogenized in sterile 225 ml tryptone soy broth (TSB) (Lab M, Lancashire, UK) supplemented with 7.5% NaCl, resulting in a first-order dilution (10^−1^). The homogenization process lasted for 1 minute at 800 rpm using a shaker, followed by serial dilution (10^−1^–10^−5^). Each dilution was plated by transferring 100 µl of diluent to three plates of oxacillin resistance screening agar (ORSA) supplemented with oxacillin (2 mg) and polymyxin B (5 mg) per litre (Oxoid, UK). The inoculum was evenly spread over the agar surface using a sterile bent glass streaking rod. Plates were kept upright until the inoculum was absorbed (approximately 10 minutes on properly dried plates), then inverted and incubated for 24 hours at 37°C. Blue-coloured colonies, indicative of presumptive MRSA, were enumerated and expressed in CFU/g. Subsequently, blue-coloured colonies from the ORSA were aseptically transferred to Baird-Parker agar plates (Merck KGaA, Darmstadt, Germany) and incubated for 48 hours at 37°C. Colonies meeting specified criteria—with an outer clear zone, displaying a buttery/gummy consistency when probed with an inoculating needle, smooth, circular, convex, 2-3 mm in diameter, moist, gray to jet-black, with an off-white margin, surrounded by an opaque zone—were selected for further purification. These selected colonies were streaked onto tryptone soy agar (TSA) supplemented with 7.5% NaCl (Lab M, Lancashire, UK) for purification and subsequently stored on Nutrient agar (Lab M, Lancashire, UK) slants at 4°C until further use. The identification of the isolates was based on a series of cultural, morphological, and biochemical tests, including Gram staining, 3% potassium hydroxide testing, coagulase, catalase, and anaerobic utilization of mannitol and glucose, following the procedures outlined by Tallent et al. (2019). The MRSA isolates underwent further characterization. A positive control in the form of *S. aureus* (ATCC 12600) was employed for reference purposes.

### DNA extraction and molecular characterization of the MRSA isolates

To resuscitate all isolates, they were placed in 5 ml of TSB after an initial 24-hour incubation at 37°C. Following this, cells were collected by centrifuging 2 ml of the incubated broth in sterile Eppendorf tubes at 5000 rpm for 10 minutes. The cell deposit underwent a washing step with normal saline after discarding the supernatant, followed by re-centrifugation at 5000 rpm for 3 minutes. The cell pellet was subsequently re-suspended in a microcentrifuge tube containing a rapid lysis buffer. This lysis buffer included the following components: 100 mM NaCl, 10 mM Tris-HCl at pH 8.3, 1 mM EDTA at pH 9.0, and 1% Triton X-100. The mixture was boiled for 15 minutes, and subsequently, it was centrifuged at 10000 rpm. The resulting supernatant was collected and stored at −20°C for future use as the template DNA. To identify *S. aureus* (based on the *nuc* gene), a polymerase chain reaction (PCR) was conducted using specific primers, as previously reported by Brakstad et al. (1992), with *S. aureus* ATCC 12600 serving as the positive control. The specific primer used for identification is detailed in Table 1 for the amplification of AMR genes, such as resistance to methicillin (*mecA*), tetracycline (*tetM*), erythromycins (*ermA, ermC*), fluoroquinolone [*aac (6′)-Ib-cr, qnrA, qnrS, qnrB*], vancomycin (*vanA, vanC*), as well as virulence genes including PVL, *tsst*-1, procedures outlined in prior studies (Monday and Bohach 1999, Martineau et al. 2000, Strommenger et al. 2003) were followed. *SCCmec* typing of MRSA isolates involved PCR amplification of *SCCmec* types 1 to V and subtype *SCCmec* (Iva to d) as described by Okuma et al. (2002), Ma et al. (2005), and Zhang et al. (2005). Specific primer sets listed in Table 1 were used. The PCR products were electrophoresed on a 1% agarose gel at 110 V for 45 minutes and visualized after ethidium bromide staining using a transilluminator (Vilber Lourmat, EBOX VX5, France).

**Table 1.** Primers used in this study.

Name	Target genes	Primer sequences (5′ →3′)	Size (bp)	References
*S. aureus*	*Nuc*	F:CGATTGATGGTGATACGGTT R:ACGCAAGCCTTGACGAACTAAAGC	279	Brakstad et al. (1992)
Panton valentine leucocidin	*pvl*	F:ATCATTAGGTAAAATGTCTGGACATGATCCA R:GCATCAAGTGTATTGGATAGCAAAAGC	433	Lina *et al*. (1999)
Methicillin resistance	*mec*A	F: ACTGCTATCCACCCTCAAAC R:CTGGTGAAGTTGTAATCTGG	163	Mehrotra et al. (2000)
Toxic shock syndrome toxin	*tsst-1*	F: ACCCCTGTTCCCTTATCATC R: TTTTCAGTATTTGTAACGCC	326	Mehrotra et al. (2000)
Exfoliative A	*eta*	F:GCAGGTGTTGATTTAGCATT R:AGATGTCCCTATTTTTGCTG	93	Mehrotra et al. (2000)
Exfoliative B	*etb*	F:ACAAGCAAAAGAATACAGCG R:GTTTTTGGCTGCTTCTCTTG	226	Mehrotra et al. (2000)
SE A	*sea*	F:TGTATGTATGGAGGTGTAAAC R: ATTAACCGAAGGTTCTGT	270	Sharma et al. (2000)
SE B	*seb*	F:TGTATGTATGGAGGTGTAAAC R: ATAGTGACGAGTTAGGTA	165	Sharma et al. (2000)
SE C	*sec*	F:TGTATGTATGGAGGTGTAAC R:AATTGTGTTTCTTTTATTTTCATAA	102	Sharma et al. (2000)
SE D	*sed*	F:TGTATGTATGGAGGTGTAAC R:TTCGGGAAAATCACCCTTAA	306	Sharma et al. (2000)
SE E	*see*	F:TGTATGTATGGAGGTGTAAC R: GCCAAAGCTGTCTGAG	213	Sharma et al. (2000)
Tetracyclines	*tet*M	F:GTGGACAAAGGTACAACGAG R:CGGTAAAGT TCG TCACACAC	406	Ng et al. (2001)
Erythromycins	*erm*A	F: TATCTTATCGTTGAGAAGGGATT R:CTACACTTGGCTTAGGATGAAA	139	Martineau et al. (2000)
Erythromycins	*erm*C	F:CTTGTTGATCACGATAATTTCC R: ATCTTTTAGCAAACCCGTATTC	190	Martineau et al. (2000)
Vancomycin	*vanA*	F:GCGCGGTCCACTTGTAGATA R: TGAGCAACCCCCAAACAGTA	314	Nam et al. (2013)
Vancomycin	*vanC*	F: ATCCAAGCTATTGACCCGCT R: TGTGGCAGGATCGTTTTCAT	402	Nam et al. (2013)
Fluoroquinolone	*qnrA*	F: TTCTCACGCCAGGATTTGAG R:TGCCAGGCACAGATCTTGAC	571	Bouchakour et al. (2010)
Fluoroquinolone	*qnrB*	F: TGGCGAAAAAATTGAACAGAA R: GAGCAACGATCGCCTGGTAG	594	Bouchakour et al. (2010)
Fluoroquinolone	*qnrS*	F: GACGTGCTAACTTGCGTGAT R:AACACCTCGACTTAAGTCTGA	388	Bouchakour et al. (2010)
Fluoroquinolone	*aac (6′)-Ib-cr*	F: TTGCGATGCTCTATGAGTGGCTA R: CTCGAATGCCTGGCGTGTTT	482	Robicsek et al. (2006)
Type I	*ORF E008*	F:GCTTTAAAGAGTGTCGTTACAGG R: GTTCTCTCATAGTATGACGTCC	613	Zhang et al. (2005)
Type II	*kdpE*	F:GATTACTTCAGAACCAGGTCAT R: TAAACTGTGTCACACGATCCAT	287	Kondo et al. (2007)
Type III	*J1 III*	F:CATTTGTGAAACACAGTACG R:GTTATTGAGACTCCTAAAGC	243	Milheirico et al. (2007)
Type Iva	*ORF CQ002*	F: GCCTTATTCGAAGAAACCG R:CTACTCTTCTGAAAAGCGTCG	776	Zhang et al. (2005)
Type IVb	*J1* Ivb	F:AGTACATTTTATCTTTGCGTA R:AGTCATCTTCAATATGGAGAAAGTA	1000	Okuma et al. (2002)
Type IVc	*Ivc*	F:TCTATTCAATCGTTCTCGTATT R:TCGTTGTCATTTAATTCTGAACT	677	Ma et al. (2005)
Type IVd	*CD002*	F:AATTCACCCGTACCTGAGAA R:AGAATGTGGTTATAAGATAGCTA	1242	Kondo et al. (2007)
Type IVh	*J1*	F:TTCCTCGTTTTTTCTGAACG R:CAAACACTGATATTGTGTCG	663	Milheirico et al. (2007)
Type V	*ORF V011*	F:GAACATTGTTACTTAAATGAGCG R:TGAAAGTTGTACCCTTGACACC	325	Zhang et al. (2005)

### The biofilm formation profile of MRSA isolates

Pure MRSA colonies were introduced into 4.5 ml of TSB and then subjected to incubation at 37°C for 18 hours. Subsequently, these cultures underwent centrifugation for 2 minutes at 12 000 rpm. The resulting cell pellets were washed and re-suspended in phosphate-buffered solution (PBS) adjusted to pH 7.2, targeting 0.5 McFarland standards. The prepared suspension inoculants were introduced into the wells of sterile 96-well polystyrene microtiter plates containing 20 ml of cell suspensions and 180 ml of TSB to investigate the adherence of Staphylococci to a solid surface, following the procedure described previously (Igbinosa et al. 2022). The negative control wells contained only TSB broth, while *S. aureus* ATCC 12600 served as the positive control. Each of the assays was performed in triplicate to calculate the mean value. Based on previously established protocols (Igbinosa et al. 2022), biofilm formation was classified as non-producing/negative (ODi < ODc), weak/poor-producing (ODc < ODi < 0.1), moderate/intermediate-producing (0.1 < ODi < 0.12), or strong producer (ODi > 0.12).

### Antimicrobial susceptibility profile of the MRSA isolates

Antimicrobial susceptibility testing was conducted through the Kirby-Bauer disk diffusion method. Aseptically, a suspension of the isolated test strains was streaked on Mueller-Hinton agar (obtained from Lab M, Lancashire, UK) using sterile swab sticks, and the corresponding antibiotic discs were aseptically positioned on the inoculated agar. The antibiotic discs used in this study were all sourced from Mast Diagnostics, United Kingdom. The disc employed includes penicillinase-stable penicillins (oxacillin 30 µg), cephems (ceftaroline 30 µg), aminoglycosides (gentamicin 10 µg), macrolides (erythromycin 15 µg), tetracyclines (tetracycline 30 µg), fluoroquinolones (ciprofloxacin 5 µg), lincosamides (clindamycin 2 µg), nitrofurantoin (nitrofurantoin 300 µg), folate pathway antagonists (trimethoprim-sulfamethoxazole 1.25/23.75 µg), ansamycins (rifampin 5 µg), phenicols (chloramphenicol 30 µg), oxazolidinones (linezolid 30 µg), and streptogramins (quinupristin-dalfopristin 15 µg). The agar plates were allowed to air-dry at room temperature for ∼10 minutes, followed by an incubation at 37°C for 24 hours. The diameter of the inhibition zone was measured using a transparent meter rule and interpreted according to recognized criteria, categorizing the strains as sensitive (S), intermediate resistant (I), or resistant (R) by the standards recommended by the Clinical Laboratory Standards Institute (CLSI 2020). For antibiotics such as glycopeptides (vancomycin 1–32 µg/ml), lipoglycopeptides (teicoplanin 4–64 µg/ml, oritavancin 0.12–0.50 µg/ml), lipopeptides (daptomycin 0.5–4 µg/ml), and oxazolidinones (tedizolid 0.25–4 µg/ml), minimum inhibitory concentration (MIC) protocols were applied, and the results were assessed following established interpretive guidelines and MIC breakpoints (μg/ml), as stipulated by CLSI (CLSI 2020). The multiple antibiotic resistance index (MARI) was calculated as previously described by Krumperman (1983). MDR profile was described as resistance to a ≥ 1 antibiotic in a ≥ 3 antimicrobial classes (Magiorakos et al. 2012).


\begin{eqnarray*}
&&{\rm Multiple\,\,antibiotic\,\,resistance\,\,index}\\
&& = \frac{{{\rm number\,\,of\,\,antibiotics\,\,to\,\,which\,\,resistance\,\,occurred}}}{{{\rm number\,\,of\,\,antibiotics\,\,to\,\,which\,\,the\,\,isolates\,\,were\,\,tested}}}
.
\end{eqnarray*}

### Virulence factor formation

#### Haemolysis of red blood cells

All MRSA isolates were cultured on blood agar supplemented with 10% sheep blood and incubated for 24 hours at 37°C. Positive hemolysis was confirmed by observing clear zones of hemolysis and a pale-yellow discoloration of the blood agar medium. All MRSA isolates exhibited beta-hemolysis. **Lysostaphin sensitivity** the sensitivity of the MRSA isolates to lysostaphin was assessed using a standardized procedure. Isolated colonies were transferred to PBS and emulsified. Half of the suspended cells served as a control, while the other half was treated with lysostaphin at a concentration of 25 µg/ml. Both samples were then incubated, and turbidity clearing within 2 hours indicated a positive reaction, suggestive of lysostaphin sensitivity. Conversely, the absence of clearing within the specified timeframe indicated a negative result. **Protease activity:** the extracellular protease activity of the isolates was assessed on TSA plates containing 1% casein. Colonies from TSB agar were suspended in Mueller-Hinton broth (MHB), with the suspension density adjusted to 0.5 McFarland standards. Samples of this suspension were inoculated onto TSA plates supplemented with 1% casein and then incubated at 37°C for 24–48 hours. Positive results were indicated by zones of clearance surrounding the colonies, indicating casein hydrolysis, while the absence of clearance was considered negative. **Hyaluronidase activity:** a stock solution of 1000 units/ml enzyme was prepared in cold enzyme diluent and further diluted to ∼6 units/ml. In a 2.0 ml reaction mix, final concentrations included 0.015% (w/v) hyaluronic acid, 150 mM sodium phosphate, and 2–5 units of hyaluronidase. A 0.750 ml enzyme solution was combined with 0.250 ml enzyme diluent and incubated at 37°C for 10 minutes. Then, 1 ml of hyaluronic acid solution was added, mixed, and incubated at 37°C for 45 minutes. Test bacterial and blank samples were mixed with acidic albumin solution and left for 10 minutes, and % transmittance was measured at 600 nm. Hyaluronidase activity was calculated using the formula provided, considering the dilution factor of the enzyme and the volume of enzyme solution used in the reaction.


\begin{eqnarray*}
&& \left( {\% T\,\,\textit{Test} - \% T\,\,\textit{Blank}} \right)\left( {df} \right)\textit{Units}/ml\,\,\textit{enzyme}\,\,\\
&&\quad = \left( {14.84} \right)\left( {ml\,\,of\,\,\textit{enzyme}\,\,\textit{solution}} \right)
.
\end{eqnarray*}

In the formula, df represents the dilution factor of the enzyme, 14.84 stands for the Sigma–Aldrich Determined Extinction Coefficient, and ml of enzyme solution indicates the volume of enzyme solution utilized in the reaction.

#### Thermostable nuclease production

The toluidine blue-deoxyribonucleic acid agar test was employed to detect nuclease production, offering specificity comparable to the coagulase test but with reduced subjectivity due to a distinct colour transition from blue to bright pink. Microslides were coated with the agar, and wells were created for sample application. Heated samples from broth cultures used in the coagulase test were added to the wells and incubated. A positive reaction, indicative of nuclease production, was denoted by the appearance of a bright pink halo extending at least 1 mm from the well's periphery. All MRSA isolates tested positive for nuclease production using this method. **Lipase activity:** the lipase activity of the isolates was evaluated using TSA plates supplemented with 1% Tween 80. Colonies from TSB agar were suspended in MHB, with the suspension density adjusted to 0.5 McFarland standards. Samples of this suspension were inoculated onto TSA plates and then incubated for 24–48 hours at 37°C. Lipases facilitate the breakdown of lipids, enabling organisms to convert lipids into smaller fragments used for various cellular processes. Lipase-producing organisms are identified by the presence of a clear halo surrounding their growth areas on the agar. **Collagenase assay:** in summary, purified isolates were cultured in 5 ml of TSB overnight. The turbidity of the broth was assessed to match the OD of the McFarland standard, equating to 10^6^ cfu/ml. Subsequently, 10 µl of the test bacteria was added to wells in a 96-well microtitre plate and adjusted to a volume of 100 µl with collagenase assay buffer. Separate wells were designated for positive and negative controls, with absorbance measured kinetically at OD 345 nm over 5 minutes and 2 hours at 37°C. Collagenase activity was determined using the formula:


\begin{eqnarray*}
&& {Collagenase\,\,activity}\\
&&\quad = \frac{\left [\left(\frac{{ - \Delta {A_{345{\rm nm}}}}}{\Delta T} Test \right ) - \left(\frac{{ - \Delta {A_{345{\rm nm}}}}}{\Delta T}Reagent\,\,background \right ) \right ] \times (0.2) \times DF}{(0.53) \times V}U/ml
.\end{eqnarray*}

In the given equation, ∆A_345nm_ represents the difference between A_345nm2_ and A_345nm1_; ∆T indicates the difference between T_2_ and T_1_; 0.2 denotes the reaction volume (ml); DF stands for the dilution factor; 0.53 signifies the millimolar extinction coefficient of (FALGPA); and V represents the volume of the enzyme (ml).

### Statistical analysis

The data obtained from this study were analyzed using SPSS version 21.0 and Microsoft Excel 2013. Descriptive statistics, such as means and standard deviations, were computed to summarize the data. One-way analysis of variance was applied to analyze multiple variables, and the Duncan Multiple Range test was conducted to detect significant differences between means. Statistical significance was determined at a probability value below 0.05.

## Results

### Incidence and density of MRSA on RTE foods

In this study, the prevalence of MRSA-positive samples in RTE foods was determined (Table 2). The detection method relied on observing the proliferation of blue-coloured colonies on ORSA supplemented with polymyxin B and oxacillin. Out of the 400 RTE food samples tested, 57 samples tested positive for MRSA, indicating a prevalence rate of 14.25%. The MRSA counts exhibited a wide range, ranging from undetectable levels to as high as 9.8 × 10^4^ ± 2.47 CFU/g, with a mean count of 8.1 × 10^3^ ± 1.46 CFU/g (Table 2). Notably, some samples exceeded the acceptable limit set by the Food Standards of Australia New Zealand (FSANZ), which is ≤10^4^ CFU/g (≤4 log CFU/g) for safe consumption. Subsequently, a total of 79 MRSA isolates were successfully identified using PCR, and these isolates underwent further screening to assess their phenotypic and genotypic characteristics.

**Table 2.** Prevalence and population density of MRSA on the RTE foods.

Type of sample collected	Number of samples examined	Prevalence of MRSA-positive samples	Mean population density of MRSA (CFU/g)
Fried rice	14	2(14.29)	8.4 × 10^2^ ± 1.41^b^
Coconut rice	12	3(25.0)	2.9 × 10^2^ ± 1.96^b^
Chicken stew	12	2(16.67)	7.6 × 10^2^ ± 1.43^b^
White rice	12	-	-
Fried beans and plantain	14	3(21.43)	4.1 × 10^2^ ± 1.72^b^
Melon soup	16	5(31.25)	6.9 × 10^3^ ± 1.44^c^
Eba	12	-	-
Banga soup	14	3(21.43)	2.1 × 10^3^ ± 1.16^c^
Starch	12	-	-
Ogwo soup	12	1(8.33)	3.8 × 10^2^ ± 0.19^b^
Okro soup	16	4(25.0)	5.1 × 10^3^ ± 2.18^c^
Goat meat pepper soup	12	-	-
Banga rice	12	1(8.33)	2.3 × 10^2^ ± 1.01^b^
Ukodo	14	1(7.14)	1.9 × 10^2^ ± 0.89^b^
Ogbon soup	14	-	-
Pounded Yam	12	-	-
Puff-Puff	12	-	-
Akara	12	3(25.0)	3.1 × 10^1^ ± 1.27^a^
Suya	16	7(43.75)	9.8 × 10^4^ ± 2.47^d^
Yam porridge	14	1(7.14)	1.2 × 10^1^ ± 0.14^a^
Roasted plantain	12	-	-
Chin chin	12	-	-
Moi moi	12	3(25.0)	2.1 × 10^2^ ± 1.19^b^
Afang soup	12	-	-
African Salad	16	4(25.0)	6.4 × 10^4^ ± 2.14^d^
Asun	12	-	-
Jellof rice	16	3(18.75)	2.4 × 10^2^ ± 1.76^b^
Snail sauce	16	4(25.0)	3.3 × 10^3^ ± 1.97^c^
Vegetable soup	16	6(37.5)	6.1 × 10^4^ ± 2.19^d^
Catfish pepper soup	12	1(8.33)	1.1 × 10^1^ ± 1.10^a^
Total	400	57(14.25)	

Legend: significant differences in MRSA counts were observed across the different types of food samples studied. These differences were statistically denoted with superscript alphabets across columns. Values with different alphabets denote significant differences, while values with the same alphabets show no significant difference.

### Virulence factors of enterotoxins and other components detected in MRSA isolates from RTE foods

**Figure 1.**
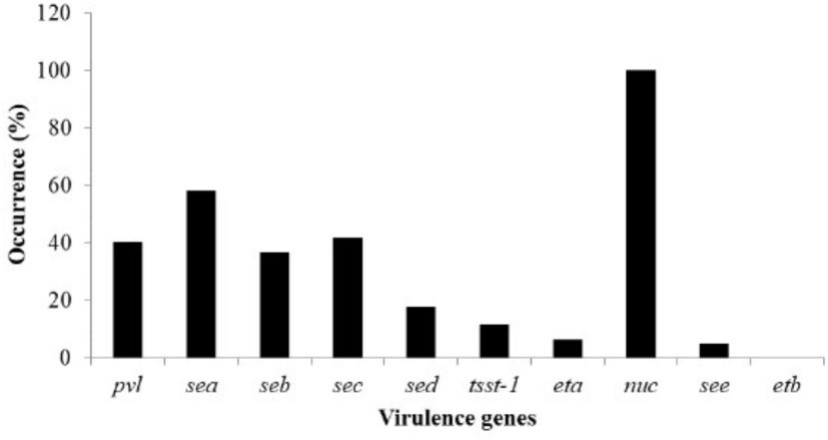
Virulence determinants of enterotoxin and other elements present in the MRSA isolated from RTE foods.

The MRSA isolates were assessed for the occurrence of enterotoxins and other virulence genes. The distribution of these genes is summarized in Fig. 1. Among the isolates, the *nuc* and *mecA* genes were universally present in all 79 samples (100%). The occurrence of other virulence genes varied: *pvl* was detected in 32 isolates (40.51%), *sea* in 46 isolates (58.23%), *seb* in 29 isolates (36.71%), *sec* in 33 isolates (41.77%), *sed* in 14 isolates (17.72%), *see* in 4 isolates (5.06%), and *tsst-1* in 9 isolates (11.39%). Notably, at least one of the virulence genes examined in this study was identified in 68 isolates (86.1%). The *sea* gene was the most commonly detected enterotoxin gene, succeeded by *sec, seb, sed*, and *see*. Enterotoxin genes were found in 58 isolates (73.42%), with 31 isolates (39.24%) exhibiting a cluster of *sea/seb/sec* genes. Moreover, the majority of MDR MRSA isolates, specifically 63 out of 66 (95.45%), harboured at least one virulence gene. Furthermore, 61 isolates (77.22%) carried multiple toxin genes, indicating a potential for enhanced pathogenicity and virulence among these strains. The exfoliative A (*eta*) gene was present in 6.33% of the isolates, while the exfoliative B (*etb*) gene was absent in all isolates.

### AMR genes and *SCCmec* of MRSA from RTE foods

**Figure 2.**
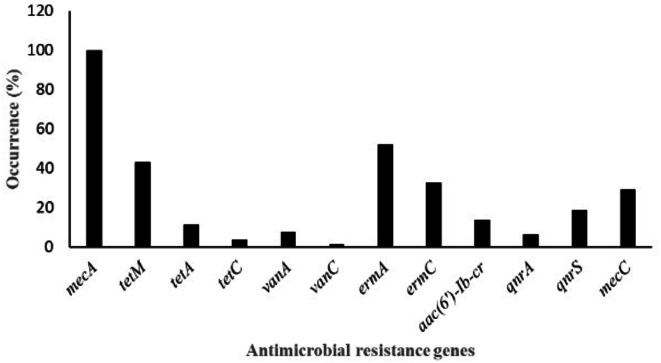
AMR genes detected in MRSA from RTE foods.

The MRSA isolates harboured various AMR genes, as depicted in Fig. 2. The proportions of these genes are as follows: *mecA* was present in all 79 isolates (100%). The *tetM* gene was detected in 34 isolates (43.04%), while *tetA* was found in 9 isolates (11.39%) and *tetC* in 3 isolates (3.8%). The *vanA* gene was identified in 6 isolates (7.59%), whereas *vanC* was observed in only 1 isolate (1.27%). Additionally, the *ermA* gene was present in 41 isolates (51.9%), while *ermC* was found in 26 isolates (32.91%). Furthermore, the *aac(6′)-Ib-cr* gene was detected in 11 isolates (13.92%), while *qnrA* was identified in 5 isolates (6.33%) and *qnrS* in 15 isolates (18.99%). The *mecC* gene was present in 23 isolates (29.11%). The *qnrB* gene was absent in all of the isolates.

**Figure 3.**
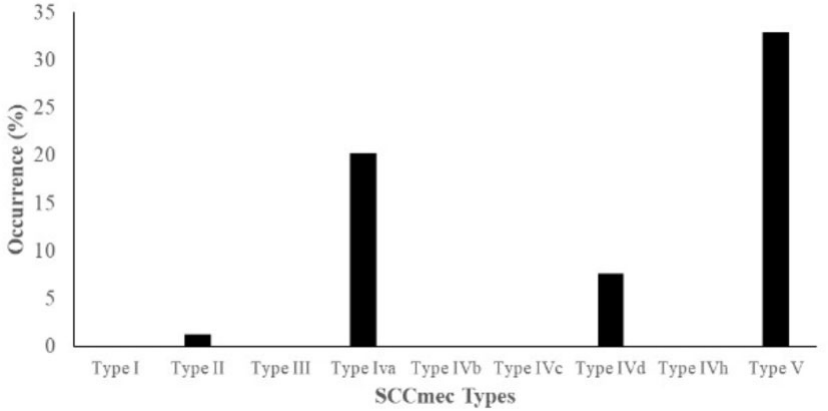
*SCCmec* types detected in MRSA from RTE foods.

Upon typing the MRSA isolates, it was determined that *SCCmec* type V was the most prevalent, followed by type Iva, with type IVd and type II being less common (Fig. 3). Among the MRSA isolates, *SCCmec* types were detected in 49 (62.03%) isolates. Specifically, *SCCmec* type II was found in 1 isolate (1.27%), type Iva in 16 isolates (20.25%), type IVd in 6 isolates (7.59%), and type V in 26 isolates (32.9%). Notably, types I, III, IVb, IVc, and IVh were not identified in any of the isolates. It is noteworthy that all isolates with *SCCmec* type V also harboured the *pvl* determinant. These findings underscore the diversity of *SCCmec* types among MRSA isolates, with type V being the predominant type encountered in this study.

### Antimicrobial susceptibility pattern of MRSA Isolates from RTE foods

Table 3 illustrates the AMR and susceptibility profiles of MRSA isolates. The MRSA strains exhibited resistance to various antibiotics, including oxacillin (100%), erythromycin (92.41%), chloramphenicol (87.34%), clindamycin (77.22%), ceftaroline (65.82%), tetracycline (59.49%), gentamicin (36.71%), trimethoprim-sulfamethoxazole (27.85%), ciprofloxacin (24.05%), nitrofurantoin (16.46%), rifampin (13.92%), quinupristin-dalfopristin (10.13%), vancomycin (8.86%), linezolid (1.27%), and tedizolid (1.26%). Notably, no resistance was observed against oritavancin, teicoplanin, and daptomycin. Conversely, the MRSA isolates demonstrated sensitivity to certain antibiotics, including oritavancin (100%), teicoplanin (100%), daptomycin (100%), linezolid (94.94%), quinupristin-dalfopristin (86.08%), rifampin (75.95%), vancomycin (75.95%), nitrofurantoin (73.42%), ciprofloxacin (58.23%), and trimethoprim-sulfamethoxazole (53.16%). These findings highlight the varying susceptibility patterns of MRSA isolates to different antimicrobial agents, underscoring the importance of judicious antibiotic use and surveillance in combating MRSA infections.

**Table 3.** The susceptibility of MRSA isolates from RTE foods to antimicrobial agents.

		MRSA isolates (*n* = 79)		
Antimicrobial class	Antibiotics	Resistant	Intermediate	Sensitive
Penicillinase-stable penicillins	Oxacillin	79(100)	NA	0
Cephems	Ceftaroline	52(65.82)	14(17.72)	13(16.46)
Aminoglycosides	Gentamicin	29(36.71)	22(27.85)	28(35.44)
Macrolides	Erythromycin	73(92.41)	6(7.59)	0
Tetracyclines	Tetracycline	47(59.49)	18(22.78)	14(17.72)
Fluoroquinolones	Ciprofloxacin	19(24.05)	14(17.72)	46(58.23)
Nitrofurantoin	Nitrofurantoin	13(16.46)	8(10.13)	58(73.42)
Lincosamides	Clindamycin	61(77.22)	14(17.72)	4(5.06)
Folate pathway antagonists	Trimethoprim-sulfamethoxazole	22(27.85)	15(18.98)	42(53.16)
Phenicols	Chloramphenicol	69(87.34)	6(7.59)	4(5.06)
Ansamycins	Rifampin	11(13.92)	8(10.13)	60(75.95)
Streptogramins	Quinupristin-dalfopristin	8(10.13)	3(3.79)	68(86.08)
Oxazolidinones	Linezolid	1(1.27)	3(3.79)	75(94.94)
	Tedizolid	1(1.26)	2(2.53)	76(96.20)
Glycopeptides	Vancomycin	7(8.86)	12(15.19)	60(75.95)
Lipoglycopeptides	Oritavancin	0	NA	79(100)
	Teicoplanin	0	0	79(100)
Lipopeptides	Daptomycin	0	NA	79(100)

Legend: NA—not applicable.

### MARI and MDR Profile of the MRSA Isolates

All MRSA isolates exhibited resistance to at least one antibiotic (Table 4). One MRSA isolate demonstrated resistance to 14 antibiotics, including oxacillin, ceftaroline, gentamicin, tetracycline, erythromycin, ciprofloxacin, nitrofurantoin, trimethoprim-sulfamethoxazole, clindamycin, chloramphenicol, rifampin, quinupristin-dalfopristin, tedizolid, and linezolid, representing 13 different antimicrobial classes, with a MARI of 0.77. Of all MRSA isolates, 66 out of 79 (83.54%) were classified as MDR, demonstrating resistance to oxacillin, erythromycin, and chloramphenicol, belonging to ≥ 3 different antimicrobial classes, with a MARI of 0.17. Moreover, 58 isolates (73.42%) were resistant to at least four antibiotics, namely erythromycin, clindamycin, oxacillin, and chloramphenicol, spanning four antimicrobial classes, with a MARI of 0.22. The MARI values among the overall MDR isolates ranged from 0.17 to 0.77 (Table 4).

**Table 4.** MARI and MDR profile of the MRSA isolates.

N^o^ of antimicrobial class	N^o^ of antibiotics	Resistance phenotypes	N^o^ of MRSA isolates (*n* = 79)	MARI
11	11	OXA^R^, CPT^R^, GEN^R^, ERY^R^, TET^R^, CIP^R^, NIT^R^, CLI^R^, SXT^R^, CHL^R^, RIF^R^	4(5.06)	0.61
7	7	OXA^R^, CPT^R^, GEN^R^, ERY^R^, TET^R^, CLI^R^, CHL^R^	22(27.85)	0.39
9	9	OXA^R^, CPT^R^, GEN^R^, ERY^R^, TET^R^, CIP^R^, CLI^R^, SXT^R^, CHL^R^	15(18.98)	0.50
10	10	OXA^R^, CPT^R^, GEN^R^, ERY^R^, TET^R^, CIP^R^, CLI^R^, SXT^R^, CHL^R^, QDA^R^	6(7.59)	0.56
8	8	OXA^R^, CPT^R^, GEN^R^, ERY^R^, TET^R^, CLI^R^, SXT^R^, CHL^R^	19(24.05)	0.44
7	7	OXA^R^, CPT^R^, ERY^R^, TET^R^, CLI^R^, CHL^R^, VAN^R^	7(8.86)	0.39
4	4	OXA^R^, ERY^R^, CLI^R^, CHL^R^	58(73.42)	0.22
6	6	OXA^R^, CPT^R^, ERY^R^, TET^R^, CLI^R^, CHL^R^	45(56.96)	0.33
13	14	OXA^R^, CPT^R^, GEN^R^, ERY^R^, TET^R^, CIP^R^, NIT^R^, CLI^R^, SXT^R^, CHL^R^, RIF^R^, QDA^R^, LNZ^R^, TED^R^	1(1.27)	0.77
5	5	OXA^R^, CPT^R^, ERY^R^, CLI^R^, CHL^R^	49(62.03)	0.28
3	3	OXA^R^, ERY^R^, CHL^R^	66(83.54)	0.17

**Legend: OXA**: oxacillin, **CPT**: ceftaroline, **GEN**: gentamicin, **TET**: tetracycline, **ERY**: erythromycin, **CIP**: ciprofloxacin, **NIT**: nitrofurantoin, **SXT**: trimethoprim-sulfamethoxazole, **CHL**: chloramphenicol, **CLI**: clindamycin, **QDA**: quinupristin-dalfopristin, **LNZ**: linezolid, **RIF**: rifampin, **VAN**: vancomycin, **ORI**: oritavancin, **TEI**: teicoplanin, **DAP**: daptomycin, and **TED**: tedizolid.

### Extracellular virulence factors and biofilm formation of MRSA from RTE foods

**Figure 4.**
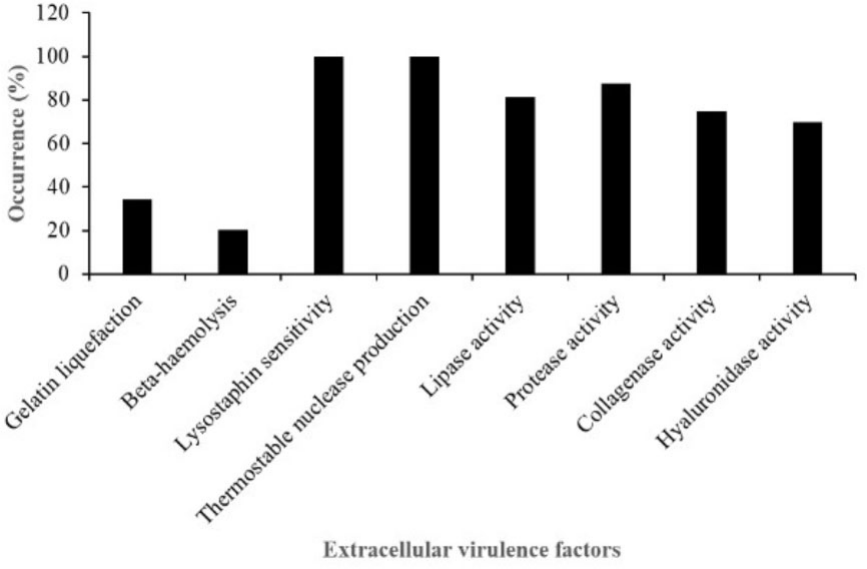
Extracellular virulence factors formation of MRSA from RTE foods.

**Figure 5.**
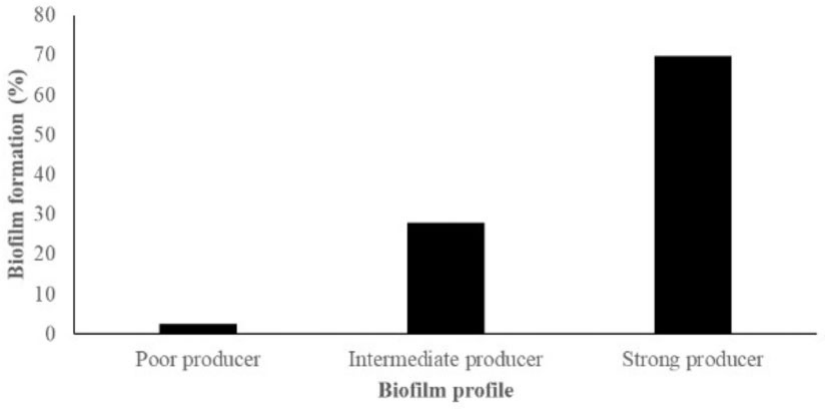
Biofilm formation profile of MRSA from RTE foods.

The extracellular virulence factors produced by the MRSA isolates are detailed in Fig. 4. The proportions of these factors are as follows: hyaluronidase activity was observed in 55 isolates (69.62%), collagenase activity in 59 isolates (74.68%), protease activity in 69 isolates (87.34%), lipase activity in 64 isolates (81.01%), and thermostable nuclease production as well as lysostaphin sensitivity in all 79 isolates (100%). Additionally, beta-hemolytic activity was detected in 16 isolates (20.25%), while gelatin liquefaction was observed in 27 isolates (34.17%). Moreover, all isolates involved in gelatin liquefaction also demonstrated collagenase activity. Regarding biofilm formation, MRSA isolates exhibited varying degrees of production: 2 isolates (2.53%) were classified as poor producers, 22 isolates (27.85%) as intermediate producers, and 55 isolates (69.62%) as strong producers (Fig. 5). Notably, none of the isolates were classified as non-biofilm producers.

## Discussion

*S. aureus*, including MRSA strains, are notably prevalent in RTE street foods in Delta, Nigeria. This underscores the urgency of addressing MRSA contamination in the food supply chain to decrease the risk of foodborne illness transmission. Our study found a 14.25% prevalence of MRSA in RTE food samples, which aligns with some studies but differs from others. For instance, while Jia et al. (2023) reported a higher prevalence (32.87%), Rajaei et al. (2021) found it in 36% of samples, indicating higher occurrence rates compared to our MRSA findings. Conversely, Subramaniam et al. (2023) reported a lower incidence (2.8%), suggesting a lower contamination rate than our MRSA study. Variability in prevalence rates across studies may result from differences in sample types, geographic locations, handling methods, and detection techniques employed. Notably, studies by Gelbíčová et al. (2022) and Mahros et al. (2021) reported MRSA occurrence rates of 2% and 83.1%, respectively, highlighting significant variations in MRSA contamination rates among studies. This variability poses significant health risks if contaminated foods are consumed without proper handling or cooking. Our MRSA count findings ranged from undetectable levels to 9.8 × 10^4^ ± 2.47 CFU/g, similar to other studies. Sobhy et al. (2024) reported counts ranging from 1.14104 to 7.4103 CFU/g, comparable to our findings. Thwala et al. (2022) found counts overlapping with our lower MRSA counts. Tonjo et al. (2022) noted that 21.8% of samples exceeded permissible counts, indicating higher contamination rates than our MRSA findings. Abolghait et al. (2020) reported counts lower than our MRSA counts but within the same range. Li et al. (2021) found lower average counts compared to our findings. Esemu et al. (2023) reported variability in contamination levels similar to ours. Mahros et al. (2021) reported a mean count within the range of our detected MRSA counts. These comparisons emphasize the variability in contamination levels across studies and food samples.

Detecting virulence determinants in MRSA from RTE foods, including *nuc, mecA, pvl*, enterotoxin genes (*seb, sea, sec, see, sed*), *eta*, and *tsst-1*, is vital for assessing the presence of MRSA in food and the potential severity of resulting foodborne illnesses (Thwala et al. 2022). These factors affirm the identity of MRSA, its resistance to methicillin, and the presence of cytotoxins associated with severe infections, heightening the risk of adverse clinical outcomes such as TSS and staphylococcal scalded skin syndrome. The detection of these virulence determinants emphasizes the importance of implementing rigorous food safety measures to minimize contamination and lower the incidence of foodborne illnesses (Igbinosa et al. 2016a,b, Ahmad-Mansour et al. 2021). Monitoring these determinants facilitates risk assessment, guides interventions aimed at curtailing MRSA transmission, and contributes to understanding MRSA pathogenicity. Conversely, research findings indicate variability in the prevalence and distribution of virulence genes among MRSA isolates. For instance, Salamandane et al. (2023) and Aksoy (2021) reported detection rates ranging from 5.3% to 57.9% for virulence genes. Pérez-Boto et al. (2023) noted that 53% of isolates harboured enterotoxin genes, while Sobhy et al. (2024) observed a 55.6% prevalence of the *se*a and *sea* genes. Wang et al. (2023) identified nine toxin genes in MRSA isolates, with 60% carrying multiple toxin genes. Conversely, Gelbíčová et al. (2022) found no tested toxin or virulence genes in MRSA strains. Furthermore, Beshiru et al. (2021) and Mahros et al. (2021) reported varying prevalence rates of specific toxin genes among *S. aureus* isolates, highlighting the heterogeneity in virulence gene profiles across different studies. Overall, our research provides insights into the prevalence and distribution of virulence genes among MRSA isolates from RTE foods. However, it is important to note that there is significant variability in virulence gene profiles among MRSA strains in other studies, which factors like geographical location, sample source, and methodology may influence.

The detection of AMR genes in MRSA isolates from RTE foods provides insights into the genetic mechanisms underlying AMR. Key genes like *mecA* confirm methicillin resistance in all MRSA isolates, highlighting treatment challenges. Detected genes like *tetM, tetA*, and *tetC* suggest potential tetracycline resistance, while *vanA* and *vanC* indicate potential vancomycin resistance, albeit at low frequencies. Genes *ermA* and *ermC* contribute to macrolide resistance, and *aac(6′)-Ib-cr, qnrA*, and *qnrS* suggest potential fluoroquinolone resistance. The presence of *mecC* indicates an alternative mechanism for methicillin resistance. Overall, diverse AMR genes underscore AMR complexity in foodborne pathogens, emphasizing surveillance and stewardship programs to preserve antimicrobial efficacy and mitigate spread (Igbinosa et al. 2016b,^1^). Understanding the prevalence and distribution of these AMR genes is crucial for assessing public health risks and guiding strategies (Igbinosa and Beshiru 2019, El-Ashker et al. 2020, Beshiru and Uwhuba 2023). Other research reveals distinct patterns of AMR gene distribution among *S. aureus* isolates. Salamandane et al. (2022) reported substantial *mecA* and *vanA* prevalence, indicating significant methicillin and vancomycin resistance. Sobhy et al. (2024) demonstrated a complex genetic landscape of AMR. Thwala et al. (2022) identified *mecC*, suggesting alternative methicillin resistance. Rajaei et al. (2021) highlighted widespread *mecA* occurrence. Mesbah et al. (2021) found *blaZ, tetK, aacA-D, ermA*, and *gyrA* as prevalent resistance genes, indicating potential MDR.

Detection of *SCCmec* elements in MRSA from RTE foods informs methicillin resistance genetics and MRSA epidemiology. *SCCmec* Type II, detected in one isolate, suggests a rare occurrence in food environments associated with HA-MRSA and increased resistance gene carriage due to its larger size. Type Iva in 16 isolates, commonly found in CA-MRSA, facilitates dissemination with its smaller size (Funaki et al. 2019). Detection of *SCCmec* Type IVd in six isolates highlights diversity among MRSA strains. Type V, found in 26 isolates, is associated with CA-MRSA and limited resistance genes, all harbouring the *pvl* determinant, indicating enhanced pathogenicity (Funaki et al. 2019). The absence of other *SCCmec* types suggests a predominance of certain types of MRSA from RTE foods. Understanding *SCCmec* distribution informs transmission pathways and public health risks, aiding antimicrobial stewardship. Conversely, other studies report different *SCCmec* distributions among MRSA isolates. Issa and Aydin (2021) found *SCCmec* type IV, differing from our findings. Wang et al. (2023) identified four *SCCmec* types, showing broader diversity. Gelbíčová et al. (2022) detected *SCCmec* cassette type V, suggesting contamination from raw pork. While our study provides an understanding of *SCCmec* prevalence and distribution in RTE foods, others highlight variability across study populations and settings.

The susceptibility of MRSA isolates from RTE foods to antimicrobials provides crucial insights into resistance patterns and treatment challenges. Widespread resistance across various antibiotic classes, including oxacillin (100%), erythromycin (92.41%), and chloramphenicol (87.34%), underscores public health concerns and limits treatment options. Observations of resistance to other antibiotics, such as trimethoprim-sulfamethoxazole, tetracycline, and clindamycin, further highlight the complexity of MRSA management. Concerningly, resistance to last-resort antibiotics like linezolid (1.27%) and vancomycin (8.86%) was noted. Stringent food safety measures and surveillance are vital to mitigate the dissemination of AMR (Beshiru and Uwhuba 2023). Conversely, diverse resistance profiles among MRSA isolates are reported in various studies. For instance, Jia et al. (2020) documented high resistance rates in MRSA strains, while Pérez-Boto et al. (2023) found variability in resistance patterns across studies, with resistance rates ranging from 22.6% to 100%. Vancomycin-resistant *S. aureus* (VRSA) strains resistant to all tested antimicrobials were documented in certain studies (Mahros et al. 2021, Saber et al. 2022). Overall, understanding these evolving resistance patterns is crucial for effective antibiotic stewardship and infection control measures.

The MARI and MDR profiles of MRSA isolates offer valuable insights into the extent of AMR and the potential for MDR, which is crucial for assessing severity and guiding treatment strategies. Notably, one MRSA isolate exhibited resistance to 14 antibiotics from 13 antimicrobial classes, indicating a worrisome level of AMR. The majority of MRSA isolates (83.54%) exhibited MDR, necessitating comprehensive antimicrobial stewardship. Multiple antimicrobial resistance index values quantify AMR extent, with higher values indicating broader resistance spectra (Krumperman 1983, Beshiru and Uwhuba 2023). Variability in MARI values among MDR isolates reflects resistance degree diversity (Magiorakos et al. 2012). The prevalence of MDR MRSA isolates with high MARI values underscores extensive AMR, urging prudent antibiotic use (Igbinosa et al. 2023). Continued surveillance of MARI and MDR profiles is vital for monitoring AMR trends. In contrast, research studies report varying MDR prevalence rates among isolates, influenced by geographical location and antibiotic usage patterns. For instance, Thwala et al. (2022) found that eight out of the thirteen isolates exhibited resistance to at least one antibiotic, while Sobhy et al. (2024) observed 88 MDR isolates. Beshiru et al. (2021) reported high resistance rates, indicating variability in MDR across studies.

The extracellular production of virulence factors by MRSA isolates from RTE foods offers insights into their pathogenicity. Enzymes like hyaluronidase, collagenase, protease, and lipase facilitate bacterial spread and tissue invasion by breaking down host components (Igbinosa and Beshiru 2015, Beshiru and Uwhuba 2023). These activities indicate MRSA's ability to invade tissues and promote infection (Mancuso et al. 2021). Thermostable nuclease production aids bacterial dissemination, biofilm formation, tissue invasion, and immune evasion (Ahmad-Mansour et al. 2021). Sensitivity to lysostaphin suggests potential therapeutic implications. Beta-hemolysin lyses red blood cells, contributing to tissue damage, while gelatinase liquefies gelatin, reflecting tissue invasion capability. Beshiru et al. (2021) found all isolates phenotypically positive for β-hemolysis. Understanding these factors is crucial for assessing infection severity, guiding treatment, and preventing MRSA spread. Continued surveillance is vital for monitoring pathogenic changes and informing interventions to control MRSA infections.

The presence of biofilms in MRSA isolates from RTE foods is crucial for food safety. Biofilms enhance bacterial adhesion, stress resistance, and antimicrobial resilience. Assessing biofilm-forming abilities in MRSA is essential to understand contamination risks (Igbinosa et al. 2023). Results reveal varied capacities among isolates, with none being non-biofilm producers. Strong producers (69.62%) pose significant contamination risks, while intermediate (27.85%) and poor producers (2.53%) also contribute, albeit to varying extents. This emphasizes the importance of sanitation and adherence to food safety regulations. Conversely, other studies show similar yet diverse biofilm patterns. Aksoy (2021) found universal biofilm formation, while Salamandane et al. (2023) demonstrated adaptability to various surfaces. Beshiru et al. (2021) reported diverse biofilm capacities, with intercellular adhesion (*ica*) genes playing a role. Saber et al. (2022) identified biofilm-forming genes in the majority of their isolates, offering molecular insights. Our focus on MRSA isolates from RTE foods highlights specific contamination risks, while broader studies on *S. aureus* explore both phenotypic and genetic aspects of biofilm formation.

## Conclusion

In conclusion, we found that MRSA contamination was present in a significant proportion of RTE food samples. These MRSA isolates exhibited a diverse array of virulence factors, including enterotoxin genes, biofilm formation capability, and AMR genes. Our study highlighted the AMR profiles of MRSA isolates concerning high rates of resistance observed against various antibiotics commonly used in clinical practice. Despite this resistance, certain antibiotics, such as oritavancin, teicoplanin, and daptomycin, remained effective against MRSA isolates, underscoring the importance of judicious antibiotic use and surveillance. Furthermore, molecular typing revealed a diverse circulation of *SCCmec* types among MRSA isolates, with *SCCmec* type V being the most prevalent. The occurrence of *SCCmec* type V was consistently linked with the carriage of the *pvl* determinant, indicating a potential link between *SCCmec* types and virulence. Overall, our findings emphasize the need for continued monitoring and control measures to mitigate the dissemination of MRSA in RTE foods and the broader community. Enhanced surveillance strategies, along with effective hygiene practices and antibiotic stewardship programs, are crucial for preventing MRSA contamination and reducing the risk of AMR transmission through food sources.

## Data Availability

The datasets generated and analyzed during the current study are available in the manuscript. Upon a reasonable request, the corresponding author can provide any other information.
